# Emerging migrant workers and aspiring capitalist farmers in the aftermath of the land rush in Ethiopia

**DOI:** 10.1007/s10460-025-10738-9

**Published:** 2025-04-22

**Authors:** Moges Belay

**Affiliations:** International Institute of Social Studies, Erasmus University Rotterdam, Gondelstraat 31, 2586 ER, The Hague, The Netherlands

**Keywords:** Land rush, Migrant labor, Oromia, Small-scale commercial farms, Social reproduction

## Abstract

I argue that the recent emergence of aspiring small- and medium-size capitalist farmers in Ethiopia is both a spontaneous and state-orchestrated from and through the spectacular land rush during the past two decades. This emerging sector that shows great dynamism in land and capital accumulation has emerged not only and not primarily from the agriculture sector, but from non-agricultural and urban sectors mainly. Furthermore, this paper shows the inseparability, empirically and analytically, of land and labor, as well as production and social reproduction dynamics in explaining the character and trajectory of social change. The role played by migrant labor in the land rush has been manifested in two interconnected ways underpinned by a singular logic and social force, that is, the capitalist commodification of land and labor, namely, outward labor migration away from the land grab sites, and the inward migrant labor inflows into the same land grabs sites.

## Introduction

### Dispossession, capital accumulation and social differentiation

In understanding dynamics of social change brought about by the land rush (Borras and Franco 2024) upon land and labor as well as production and social reproduction dynamics in peasants and pastoralist societies (Scoones 2021), theoretical concepts of dispossession, capital accumulation and social differentiation are relevant. There are various competing notions of peasant families regarding the production and social reproduction role of labor. For Chayanov (1966), peasant families are self-reliant in terms of the labor required for agricultural production and social reproduction among their members. According to him, since they do not hire outside labor to make profit from what they produce, the peasant families are therefore distinct from capitalist firms. From a Marxist analytical lens, land and labor dynamics are more complex, and property relations in peasant societies can belong to three possible categories of farm households in terms of land and labor relations (Patnaik 1979). The first is the class of semi-proletariats who are the majority of the small-scale producers that are characterized by limited access to land, inadequate consumption, and surplus labor. The second category includes middle peasants who can produce enough food for their consumption needs. They have adequate access to land, and higher labor productivity, which is defined as the quantity of output produced by a given unit of labor per limited work time. The third one is the class of rich peasants who have access to sufficient land and hire outside labor to meet their consumption requirements as well as for production of surplus (ibid.).

Agrarian change in contemporary rural societies is associated with processes of social differentiation, which has influence conditions of labor supply and patterns of its mobilization (Cousins 2025). As land, nature, labor and subsistence started to be commodified in the countryside, peasants have been exposed to the process of class differentiation. Henry Bernstein argues that contemporary peasantry signifies the emergence of ‘petty commodity producers’ as capitalists with little profit on the one hand, and laborers with little control over the terms and conditions of selling their labor on the other hand. He suggests that “as a result of class formation, there is no single “class” of “peasants” or ‘family farmers’ but rather differentiated classes of small-scale capitalist farmers, relatively successful petty commodity producers and wage labor” (Bernstein 2010, p. 4; see also Harriss-White 2023).

For Marx, peasants were to get expropriated and become separated from their means of production and transformed to workers for capitalist relations to emerge (Marx 1976; org.1867). This was explored further by both Kautsky (1988, org. 1899) and Lenin (2009) who considered capitalism as a dispossessing system that significantly transforms peasantry through consolidation of capitalist production relations in agriculture. For Lenin (2009), differentiation of the peasantry and their transformation into property-less workers shows the ruin of the peasantry, and the few who resisted dispossession could, under certain condition, be transformed into capitalist farmers. This process would eventually result in the disappearance of the peasantry. Kautsky (1988, org. 1899) argues that the capitalist process of agrarian transformation altered the peasantry as way of life, both with in agriculture and even outside agriculture. Nevertheless, he contends that peasants will not completely disappear because owners of big capital would want many of these land-owning peasants to continue to have land for their social reproduction; this could allow them to be able to accept cheaper wages for the work they do for capitalist enterprises.

Following the discussion above, the contemporary peasantry is subject to various forms of dispossession and displacement due to different economic and extra-economic forces that grabbed its means of production and social reproduction, principally land. The first is “compulsion from above,” where peasants are exploited in various ways by coercion or extra-economic forces that are directly imposed on them by the state or state-affiliated institutions (Mamdani 1987, p. 198). It is basically resource grabbing and displacement of local people, where these powerful elites employ coercive force or a threat of using it, including the military, police and courts. The second is “compulsion” from below, which is mainly due to market forces, wherein peasants engage in unfair and exploitative market relations with others who accumulate capital within the same context of commodity relations (ibid.). In this case, compulsion is not directly by any political force; rather it is due to a given socio-economic context with unequal relations into which peasants enter ‘voluntarily’ to reproduce themselves. Land market transactions that facilitate transfer of land to commercial farms are mechanisms of compulsion from below, leading to the dispossession of small-scale producers, peasants and pastoralists, from their land and accumulation by the dominant capitalist class.

### Land, labor, production, and social reproduction

Informed by concepts of dispossession, accumulation and differentiation discussed above, it is relevant to employ political economy concepts on the dynamics of land and labor as well as production and social reproduction that are, in turn, fundamentally important in understanding the broader social change that the land rush may, or might already have, set forth in peasant and pastoralist societies. Compared to the classical primitive accumulation that Marx (1976; org. 1867) explained in relation to the English enclosures, which was mainly due to the alienation of peasants from their land as means of production, accumulation during neoliberalism also includes diverse forms of expropriation in other sectors of the economy in which the means of production and social reproduction of the working people are expropriated by the dominant socio-economic classes (Shivji 2017). Social reproduction is a process of creating and maintaining social ties through various forms of interactions that involve the provision of care and support (Fraser 2014). Shivji theorizes the concept of working people addressing producers in all sectors, including “peasants and pastoralists, proletarians and semi-proletarians, street hawkers selling consumer goods and peddlers selling cooked food, operators and repairers in backyard workshops” (Shivji 2017, p. 11). This has resulted in the sharp decline in relative percentage of iconic self-sufficient peasants and full-scale proletariats, and the rise of semi-proletarian class.

Bernstein (2010), loosely referring to a similar category observed by Shivji, argues that these are working people who combine various sources of livelihoods in order to survive; he calls this broad category as ‘classes of labor’. Bernstein identified four types of labor regimes, which he defines as “different methods of recruiting labor and their connections with how labor is organized in production (labor processes) and how it secures its subsistence” (2010, p. 53). The first is, what he called “forced labor”, where classes of labor, either separated from means of production or not, are supposed to provide labor in the form of slavery, tributes/tax, or mandatory service. The second is semi-proletarianization which refers to the process of formation of classes of labor who are not completely disposed of land or separated from other means of production. The third is, the process of petty commodity production which refers to small-scale production of commodities for subsistence by combining both capital and labor. The fourth one is full proletarianization of producers (e.g., farmers) through complete separation from their previous means of production.

These processes of getting separated from the means of production and social reproduction (land), and the emerging diverse ways in which working people survive are at the heart of the political economy of the land rush and its consequences. Due to its scope, the land rush has the potential to extensively restructure agrarian economies and livelihoods, as well as disrupt the existing land regime. Referring to its broader nature with the involvement of varied actors, the contemporary global land rush is defined by Borras and Franco (2024, p. 3) as “a chaotic, relatively short-lived, historical juncture marked by a sudden surge in demand for land, accompanied by an extremely speculative and competitive, often violent, and convulsive transition from one set of rules on commodity and land politics to another.” They further explained that “it refers to that insurgent moment when the prevailing ‘land regime’ is seriously challenged but not yet fully replaced by a new regime” (ibid.).^1^

The extensive displacement caused by land grabs has been validated by numerous studies (Hall 2013; Hall et al. 2015; Regassa et al. 2019; Tura 2018; Makki 2014). But the claims on the employment potential by these investment projects for the local people were exaggerated; the actual job generation tends to be far below the earlier hyperbolic claims (Li 2011; Gyapong 2020; Zaehringer et al. 2021; Bélair et al. 2024; Hall et al. 2017). The issue of labor then deserves more empirical attention, heeding to call by earlier scholars who have observed that labor is a largely ignored issue in the land grabs literature (Li 2011; Oya 2013). As argued by Li (2011), when the land is needed but the labor is not, often the result is the expulsion of villagers from the land. The general assumption is large-scale land deals that employ large-scale and industrial production system will not need any significant amount of labor. But what if the emerging capitalist enterprises actually needed land and labor? What happens then to the labor question? This is a relevant question because as it turns out, large-scale land deals and capitalist enterprises are not the only trajectory in land grabs. There are also small and medium enterprises that have emerged from the land rush (Jayne et al. 2016, 2019; Hall et al. 2017).

Any discussion about the politics of land investments is rather empty without any serious discussion about labor, whether the latter means labor displaced by or labor attracted to land investment sites. As Oya (2013, p. 1547) has emphasized, one of the possible impacts of the land deals is “the crisis in the social reproduction of labor because people leave (or are economically forced to leave) agriculture without their labor being absorbed elsewhere in the economy”. Subsequently, those displaced from the land become part of the already huge army of global reserve labor, or relative surplus population, or what others call class of the precariat (Davis 2006; Standing 2014; Li 2011). The social and ecological risks and uncertainties of agrarian societies have been generally worsened by the processes of restructuring nature through capital and labor relations which ultimately meet the interest of capital; meaning, labor and nature/land relations as Shattuck and colleagues (2023) explain. But changes in agrarian capital accumulation and labor relations cannot be effectively examined by focusing on just land size, as there is not necessarily a direct and positive relationship between land size, and the capital and labor utilized by a specific mode of production (Oya 2013). For example, the classification of ‘big’ and ‘small’ farms in Africa depends on various contextual factors including agricultural practices, crop types and the existing dominant social systems (ibid.). Although Bernstein (2010) contends that agrarian capital accumulation is limited to large-scale commercial farming, it can also develop on farms of any size. Therefore, since whether to hire outside labor is determined by “the degree of farm intensification, crop choice, and available technology”, a range of labor relations may be reflected in a certain range of farm size (Oya 2013, p. 1552). This labor question is at the heart of the challenge of understanding the meanings and implications of the contemporary land rush in Ethiopia and what its implications for ordinary working people and society are.

Small and medium farms emerging from the land grabs have not been given sufficient attention in the literature, although these have been emerging steadily throughout Africa (Hall et al. 2017; Oya 2007). Some of them are ‘middle farmers’ with significant access to land and other resources, which helped them to emerge as new forms of capitalist class (Hall et al. 2017). Most of them acquire land through various forms of ‘pin prick’ land deals. These are everyday forms of land transactions that have received less attention from the media and the academia as they involve small plots of land per transaction and do not take place through high-level memoranda of agreement with governments (Borras and Franco 2024; Wolford et al. 2024).

The scale of social reproduction at household level also significantly shapes social differentiation and emerging class dynamics in peasant societies (Deere and de Janvry 1979). In capitalist societies, most of the social reproduction tasks, though not all, are undertaken in the form of unwaged labor that occurs outside the market, including in households, communities, and other institutions such as schools and childcare centers (Fraser 2014). This conception of social reproduction is essential in understanding labor beyond unpaid domestic duties that are necessary for continuity of social relations, not merely biological reproduction and childrearing (Bhattacharya 2017). The distinction between social reproduction and commodity production is deeply historical and gendered (and generational), linking reproduction to women and production to men (Fraser 2014; White 2020). Such longstanding notion of division of labor is the main foundation of women’s and young people’s subordination in modern capitalism (ibid.).

As noted by Mezzadri and colleagues, some studies conducted in the Global South (see also Çelik 2023) show that in the processes of rural transformations and proletarianization, women have become in charge of crucial roles in both production and social reproduction process, representing new “classes of extractive labor” (Mezzadri et al. 2024, p. 4). Therefore, social reproduction as analytical tool helps in both addressing the critical strategic gender issues of the current society and comprehending its historical roots as well (Mohandesi and Teitelman 2017). Land and agriculture continue to have key role in both production and social reproduction tasks for the majority of rural households in the world today (Cousins 2025). Therefore, the traditional notion of land access, which is dominantly about land’s location in production, should be reframed into a broader perspective that equally considers both the production and social reproduction role of land, and production and social reproduction not as separate spheres (Borras and Franco 2025b).

Studies have been conducted on small-scale commercial farms in Ethiopia (Yimam et al. 2022; Afework and Endrias 2016; Girma and Kelil 2021). However, the focus of these studies was mainly on how these small-scale investments helped in commercializing the agricultural sector and increasing the productivity of commercial crops. These studies give little, if any, attention to changes in land and labor regimes associated with these projects, and these were not studies in the context of the consequences of the land rush. Taking the case of investment boom in the Upper Awash valley of Oromia region, my paper examines the consequences of the land rush in the co-constitutive processes of the emerging small and medium capitalist farms and internal labor migration. The study was conducted in A-1 kebele^2^ of 1-A woreda^3^ in the East Shewa zone, where there is continued land and investment boom since the land rush. Empirical data was collected from multiple sources during the fieldwork conducted in 2022–2023. From the host community a survey of 170 households and a focus group discussion was conducted. A focus group discussion and a survey of 120 participants were also conducted among migrant farm workers. Moreover, in-depth interviews with various actors were carried out, including 25 government officials at regional, woreda and kebele levels as well as 10 investors involved in these small-scale commercial farms.

The paper has five sections, starting with this first section that covers relevant basic concepts and arguments. Section two presents a short summary of government strategies on the expansion of commercial agriculture by smallholders as well as large-scale foreign and domestic investors, which also partly contributed to the flourishing of the small-scale commercial farms. Section three provides evidence-based critical analysis of the rise of capitalist farmers and businessmen involved in small-scale commercial farming, with a particular focus on the Upper Awash valley. Section four discusses the labor migration from other areas due to these emerging commercial farms and its implications on production and social reproduction of land and labor. In the final section, the paper concludes highlighting the potential implication of these findings for the current critical research on the land rush and relations of labor.

## The land rush: the shift towards large-scale land investments

The land rush was partly facilitated by the central state’s objective of promoting foreign and domestic large-scale land investments alongside domestic small- and medium commercial farms, although it was the former that has attracted media and academic researchers’ attention during the past two decades. After the Ethiopian People’s Revolutionary Democratic Front (EPRDF) took power in 1991, the government focused on the expansion of commercial farms and came up with various policy directions for implementation. Agricultural Development Lead Industrialization (ADLI) was one of such earliest strategies that aimed at increasing productivity of smallholders through creating better access to agricultural inputs and extension service (Zenawi 2017; Rahmato 2014; Berhanu 2016). The government’s main goal in such a strategic shift was to achieve structural transformation in the peasant agriculture, substituting the command economy with a market-driven economic system (MoFED 1993). The government’s effort to commercialize smallholder agriculture resulted in significant changes in the land use policy, restructuring agricultural extension services, and setting institutional changes at local level to encourage more successful farmers (Fantini 2015). To create access to financial services for smallholder producers and thereby commercialize their production, expansion of rural microfinances was another political commitment that was promoted by issuing the Micro-Financing Business Proclamation No. 626/2009 (FDRE 2009). However, the technical and financial supports provided to smallholder producers did not bring about significant improvements in production that could supply as per the demand of the rapid population growth (Diao 2010).

The sectors’ sluggish change, therefore, compelled the government to shift its focus towards the expansion of large-scale commercial farms by private investors, with the assumption that such investments would have greater impact in commercializing the sector rapidly (Lavers 2012; Rahmato 2011; Abbink 2011). Consequently, various rural development policies and rural land laws were introduced in the early 2000s in order to facilitate strategic shift from small-scale peasant farms to large-scale capitalist farms by private investors. Among these measures, the most remarkable ones are the Rural Development Policy and Strategies of 2003 (MoFED 2003), and the Plan for Accelerated and Sustainable Development to End Poverty (PASDEP) (MoFED 2006), which was implemented from 2005/2006 to 2009/2010. In order to attract private investors to the sector, the government also put in effect various incentive packages since 2002, implementing investment proclamation number 280/2002 (FDRE 2002).

The grand development plans have also boldly emphasized the promotion of large-scale agricultural investments. In the first Growth and Transformation Plan (GTP1), which was implemented in 2011–2015, the government sought that large-scale commercial farming would contribute to the achievement of the Millenium Development Goals by 2015 (MoFED 2010). In this plan the government has envisioned that the country would become one of the middle-income countries by 2025). To this effect, the government has planned to transfer 3.3 million hectares of land to both foreign and domestic investors for expansion of large-scale commercial farms (MoFED 2010. Besides provision of land, it is stated in the GPT1 document that the government would mobilize cheap labor from areas with surplus population as well as construct basic infrastructures to facilitate the large-scale investments (MoFED 2010). Large-scale agricultural investment by the private sector continued to be key strategies for development in the second Growth and Transformation Plan (GTP2), implemented in 2016–2020 (NPC 2016).

The land rush in Ethiopia reached its peak in 2008, driven by the already existing government efforts combined with the need for various responses to the global crisis of food, fuel, and energy, as well as a growing demand for land and natural resources by emerging industrial economies (Cotula 2012; Taylor and Bending 2009; Zoomers 2010). Although the government claimed that these lands targeted for large-scale agricultural investments were ‘empty’ lands, most of these areas are homes for various ethnic minorities and pastoralists. Several critical studies show that these areas were used by these indigenous inhabitants for various activities such as grazing, shifting cultivation, and other sociocultural activities (Gill 2015; Hajjar et al. 2020; Timko et al. 2014; Moreda 2017; Moreda and Spoor 2015; Abate 2019; Labzaé 2016; Nalepa et al. 2017; Gebresenbet 2016; Wubneh 2018). Despite the overstated claims about their supposedly development role, these large-scale land investments resulted in various social, economic, political, and environmental problems. For example, such investment project in Ethiopia have led to land dispossession, displacement and outmigration of local communities, worsening the problem of unemployment (Alamirew et al. 2015; Hajjar et al. 2020).

## The land rush and the emerging agrarian capitalists from below

Earlier, Oya (2013) pointed out that the share of large-scale land investments by foreign companies in the rise of agrarian capital in Africa might be relatively smaller compared to that of investments by domestic owners of capital, at least in aggregate terms. My study reinforces this observation. I do not have definitive quantitative evidence to show how much and which type of investment, but at least my study shows that the aggregate total of land investments by domestic investors is definitely significant. In classic agrarian studies, social differentiation allows some of the middle and rich farmers to accumulate from below and become full-scale capitalist farmers (Marx 1976; org. 1867; Lenin 2009; org. 1964). But in my study, accumulation from below and the social force constituting the emerging capitalist farmers do not seem to have overwhelmingly come from the agricultural sector, as many were in fact from urban, non-agricultural sectors. In addition to the land accumulation by a few middle and rich farmers, the emerging aspiring capitalist farmers come from a class of urban wealthy and influential individuals, such as businessmen, assorted types of entrepreneurs, government officials, and politicians. These emerging aspiring agrarian capitalists are engaged in accumulative production processes, primarily depending on: cheap land leases, cheap migrant labor, sharecropping arrangement in some instances, and concessions in taxes. In sum, the entrepreneurs in the Upper Awash valley of Oromia region can be categorized into two based on their origin. The first group comprises farmers from the community who had better access to productive resources and the capital that helped them to transform from small-scale farming to medium-scale commercial farming. These farmer investors started producing surplus for sale and began to diversify their products by growing other types of cash crops. The second category includes the businessmen and bureaucrats from urban areas who went out to the rural areas to invest in commercial farming. It is not all great and easy for these entrepreneurs either. As far as support from the government is considered, these local investors claimed that they are excluded from various incentive packages by the government, which are easily accessible for large-scale corporate level foreign and domestic investors.

The government has always targeted both foreign and big corporate investors as well as domestic prospective medium to large farmers as beneficiaries of state-driven land reallocation program. This was the two-pronged intention of the state. But there were also instances in which small- and medium-scale farmers benefited substantially from the spillover effects of these institutional and structural changes. For example, to facilitate the transfer of land for corporate level investments, the government implemented several strategies and pursued multiple legal mechanisms. In accordance with this, Oromia regional government has adopted a decree that allows farmers to autonomously rent their land to investors for a period of 15 years, which should be renewed every five years. Such legal framework has created an opportunity for the emerging rural entrepreneurs to rent land from smallholder farmers by providing higher rates of payment compared to the amount offered even by the corporate-level investors. In my case study sites, farmers feel that leasing their lands to domestic investors this way is a better option than if the government takes over their land and reallocate it to large-scale land investors, a process that has occurred nationwide in which they receive little compensation. Thus, a direct domestic investor lease contract with them, despite being for a quite long period of 30 years, is better. This is thus how many entrepreneurs accumulated land from farmers who owned smaller farmland that are suitable for cultivating cash crops such as onions, potatoes, tomatoes, and fruits like watermelon and strawberries. In many ways, this was even a ‘life-saver’ because for years of using chemicals in their own plots, the only way for them to go on is to continue using chemicals (fertilizers, pesticides) that has resulted in two negatives: it has become financially unsustainable, and it has become ecologically unsustainable. As noted by Shattuck and colleagues, “cash crop agriculture, pesticide dependence, hybrid and transgenic seeds, and chemical fertilizers are known to increase the risk of debt and land loss for small-scale farmers” (Shattuck et al. 2023, p. 494). Others have pointed out about what they perceive of possible spillover effect of adjacent intensive chemical use by capitalist farms that also affected their traditional subsistence-oriented farming. As a result, the decline in the productivity of their farmland also added yet another reason to just rent out their land to these domestic investors. It is like a snowball effect in favor of the domestic entrepreneurs in terms of land accumulation through small-scale basis at a time.

There is no clear government census data about many of the social processes of dispossession, linkages between production and social reproduction, and connections between land and labor that I aim to explore in this paper. Thus, I carried out a household survey. The primary data collected from the sample households in kebele A-1 shows that about 53% of the small farmers have rented out at least one of their plots to the domestic entrepreneurs who in turn become the emerging aspiring capitalist farmers. The respondents reported that the primary factor for renting out their lands was fear of expropriation by the government for low compensation. This shows choosing a ‘lesser evil option’, in a way. The second important factor mentioned by the participants was lack of financial capacity to invest in commercial farming, which requires more capital to purchase agricultural inputs and machinery. This is related to the respondents’ point that they rented out their land due to its decreased productivity as well as lack of water, which are mainly due to the impact of the chemical farming by the big flower farms and other medium- and small-scale commercial farms. Some smallholder farmers also expressed that they were compelled to rent out their lands since all other farmers in their surroundings have rented their land to investors. This is like a ‘crowding effect’. They believe it would be challenging, if not impossible, to farm and produce subsistence crops such as *teff* in the middle of such chemical farming of unfamiliar crops by the investors.

In addition to the institutional changes, the government has also built basic infrastructure, including roads, water supply, and electricity, to create favorable investment climate for large-the scale investment projects. These government initiatives have also contributed to the expansion of commercial farms by these emerging entrepreneurs. Regarding this, one of these domestic entrepreneurs said:

*Previously, before the expansion of the large flower farms and other investments, there were not adequate roads and transportation to the urban centers. But now the government has constructed suitable roads that make it easy for us to transport our farm products, such as perishable vegetables and fruits, to markets in the major cities, including Addis Ababa and Adama.*

Interestingly, these domestic entrepreneurs explained that they have obtained crucial knowledge regarding the application of modern farming technologies and inputs from some of these successful large-scale projects (the use of tractors, maintenance of tractors, the use of chemicals and so on). This also includes learning from the failures and achievements of the large-scale investment projects during the land rush. Again, it is not to say that everything was straightforward and easy for these entrepreneurs and big investors. Several foreign and domestic investors who had acquired land for various large-scale investments were unable to pursue the proposed projects as planned for various reasons, and from these entrepreneurs draw lessons. On one hand, they have been able to identify and avoid the constraints that led to the failure of these previous projects, which may include social, political, or ecological factors. On the other hand, the failure of these large-scale projects has created an opportunity for those emerging investors to expand their investment in commercial farming because they now have access (mainly by renting in) to the land that was previously held by these unsuccessful investors. One of the interviewees provided further explanation on this as follows:

*Compared to the previous investors or those who started investing ten years ago, we have a better understanding of the challenges associated with investing here and how to deal with them effectively. Indeed, we learned these lessons from the experience of these earlier investors.*

The incidence of several ‘failed land deals’ in these areas has also created opportunity for these emerging local investors to acquire land in various ways. As argued by Borras and colleagues (2022a), the so-called ‘failed land deals’ are considered unsuccessful just within the framework of the investor’s stated commitment to establish agribusiness ventures. Nevertheless, these ‘failed land deals’ ultimately result in new land deals with different investors, leaving the local people dispossessed and unable to reclaim their lost land which is rather returned to the government land bank (Borras et al. 2022a). In many cases, the Ethiopian government has not returned investment lands to the local communities from whom they expropriated the lands. Instead, these lands were reverted back to the land bank of the government. From there, they reallocated these to new investors, including the emerging small and medium-scale capitalist farmers described earlier. According to an important official in the investment bureau of Oromia region, the regional government has undertaken various reforms in relation to land investments since change of administration in 2018. He explained that “*we have cancelled the land lease contracts of several investors who failed to operate as per their agreement, and the land was distributed to other investors who have already started investing in it.”*

There is yet another stream through which the emerging capitalist farmers get their land: re-leasing lands through the large-scale land investors and the Ethiopian Development Bank (EDB). Some big land investors did not manage to pursue the capitalist enterprises they have originally planned, for various reasons. But they hold long-term lease contract at concessionary rental fees from the government. Instead of surrendering the land to the government, some of these investors started to retail land leases to smaller entrepreneurs. There is a regional regulation^4^ stating that the investors may rent out such leased land to other investors, provided it is for the same purposes and within the time frame specified in the lease agreement with the government (ORNS 2012). Moreover, some investors (foreign and large, domestic and small/medium) used their leased lands as collateral to secure loans from EDB– again, part of the overall strategy of the government to support commercial agriculture. But for various reasons, many of these investors defaulted on their payments to EDB, and the EDB took over these lands. Later, EDB would re-lease these lands to new domestic small- and medium entrepreneurs.

The expansion of mechanized large-scale commercial farms in the Upper Awash valley has also created an opportunity for the emerging agricultural entrepreneurs to get access to various modern agricultural technologies, including tractors, water pumps, and harvesting machines. One of the interviewed local investors stated that “*the big investors in this area are supportive. Most of them own advanced agricultural technologies, which they also rent to other small-scale investors at an affordable price*.” The rise of tractor rental market has been quite significant, although many entrepreneurs also buy second-hand tractors. The land rush has also created an opportunity for those emerging local investors to start investing in joint ventures in collaboration with foreign investors, or investors from the diaspora. In line with this, one of these local investors mentioned that he began as a local partner with a diaspora investor to invest in large-scale maize production. He explained his partnership as follows:

*I was in charge of finalizing the process of obtaining the investment license from Ethiopian Investment Commission and managing the farm, while my partner from Australia contributed the investment capital of 500*,*000 Birr. We started maize farming on a 10*,*000 hectare of land that we leased from the government.*

Many of these agricultural entrepreneurs also invest in other sectors, such as hotels, education, transportation, manufacturing, and other business activities. Some of the informants explained that they combine their assets from different sectors and use their products from one sector as inputs for the other. According to them, this strategy helps them to maximize their profits from the investments and to minimize risks. For example, they use their hotels and manufacturing plants as collateral to take loans from banks, which they in fact use to invest in the agricultural sector. Conversely, they also use their agricultural products as inputs for their hotel services and agro-processing plants. In line with this, a businessman in Adama town explained that “*presenting my hotel as collateral, I took a loan of 10 million Birr from the development bank of Ethiopia, which I used to expand my investment in commercial agriculture*.”

The Ethiopian government supports the expansion of medium- and large-scale land investments through financing. For example, the government adopted the Micro-Financing Business Proclamation No. 626/2009 (FDRE 2009) to enhance financial services to smallholder farmers through expansion of rural micro-finance institutions. The regional governments are the main shareholders of the major regional-based micro-finance institutions which are also politically affiliated with the regime and used to mobilize the rural population (Fantini 2015). One of such type of micro finance is Oromia Credit and Savings Share Company (OCSSCO), which is the largest loan provider to smallholder farmers, investors, and businessmen in the region. According to the informants, this microfinance is a key source of finance to invest in small-scale commercial farming, cattle fattening, and dairy production. Most of them reported that they have taken loans from this institution, at least once, to finance their investments in the agricultural sector. The institution has a variety of credit packages that range from small-scale loans that does not require a grantee to huge capital loans that can be offered up on presenting a collateral such as land. Farmers and investors can take-out these loans either in groups or individually based on the type of loans they want. Although the federal rural land use and administration proclamation prohibits the use of rural land as collateral in any loan agreement in general, the regional land law of Oromia allows smallholder farmers to use their farmland as collateral to take loans from microfinance institutions. A farmer who is currently investing in production of commercial crops described as follows how he benefited from such financial services to expand his investment.

*The loan I obtained from Oromia Credit and Saving Institution was crucial for me to start investing in commercial farming. When I started this investment in 2012, I first took a loan of 50*,*000 Birr, which helped me to rent a plot of farmland from my neighbor. I merged this land with my own land and purchased some inputs to increase production. I was successful in my first investment and hence managed to pay back the loan on time. After four years, in 2016, I took another loan of 200*,*000 Birr from the same institution. I added some money from my own capital and purchased a tractor, which has significantly expanded my investment. I am also getting additional income from renting the tractor to other farmers and investors.*

In short, the rising sector of small- to medium-size agrarian capitalists in Oromia does not strictly follow the classical social differentiation-induced process of land and capital accumulation from within the agriculture sector. Instead, the bulk of those able to accumulate land and capital is from outside the farming sector, mostly from urban centers. Their emergence has elements of both spontaneous dynamism and state orchestration, all emerging from out of the land rush. One key element that give rise to this emerging sector, and enabling its land and capital accumulation afterwards is the availability of cheap migrant labor, or rather, having access to the social categories that Shivji (2017) calls ‘working people’ or Bernstein (2010) calls ‘classes of labor’. We now turn to this topic in the next section.

## Migrant labor, production, and social reproduction

The land rush and the continued land grabbing in these areas have resulted in a population movement dynamic where there is outmigration and immigration of labor force occurring simultaneously at the same time and at the same place. As discussed in Sect. 2, the flower farm and other large-scale agricultural investments in the Upper Awash valley of Oromia region have caused massive population displacement and outmigration of the productive labor force to urban centers, looking for better wage labor in the other sectors. About 68% of the respondents in the household survey indicated that there was at least one household member who left to other areas due to various factors, including shortage of farmland, lack of access to employment opportunities, decreased income, and displacement by these large-scale investments. Only in a few cases, displaced smallholder farmers work as daily laborers on these investment areas to generate income that is insufficient to purchase enough food for their families.

Who then work for the emerging capitalist enterprises arising from the land grab sites? The short answer is: migrant workers from poorer regions of Ethiopia. Rural villagers from other regions of Ethiopia with a relatively more severe shortage of land (themselves also dispossessed through various social processes) and lack of job and livelihood opportunities migrate to these investment areas in the Upper Awash valley to work in the emerging small- and medium-scale commercial farms often for meager wages. A typical small- and medium scale capitalist farm terms to be labor-intensive in its production system, but since the local labor displaced by land accumulation processes migrated out, the emerging capitalist farms have to find labor supply from elsewhere. The main source of cheap migrant labor are the Southern Nations and Nationalities People (SNNP), the Sidama region, and the Central Ethiopia region^5^. These areas are characterized by high population density and shortage of farmland that led to a large surplus labor force.

According to an important official at the office of labor and social affairs of the study woreda in Oromia, thousands of seasonal migrant farmworkers come from these regions (i.e. SNPP and others) and work as daily laborers on these small-scale commercial farms. He also confirmed that despite the pressure from the office of labor and social affairs on investors to prioritize the local Oromia workers, most of the Oromia youth do not want to work as laborers in these farms because they consider the wage is too low to meet their subsistence needs. He stated that “*the office of labor and social affairs can intervene only when there are issues arising in employer and employee relations, such as the violation of the labor contract, and when there are complaints by workers regarding safety issues and other working conditions.*” He also added that “*there is not a minimum threshold labor wage set by the government, and hence it is just based on the negotiation between the parties, which is subject to the free market system*.”

A labor inspection expert in the office also explained that “*now a days, the [Oromia] youth in rural areas are becoming less interested in engaging in the traditional farming practices and more attracted to the urban lifestyles, which also contributes to the outmigration of the youth to the nearby urban centers*.” He also emphasized the contribution of the recent investment boom and land market transactions, which encourage people to rent out their land for investors and migrate to urban areas for additional cash income from working as wage laborers. Labor experts in the office perceive such labor migration as a positive development, despite the absence of better employment opportunities and proper regulation against labor exploitation. This labor inspection expert says that “*the migration of the local people to the nearby urban areas allows those landless to attempt other income-generating options as well as bring modern lifestyles and new technologies to the rural areas*.” The office of labor and social affairs, therefore, remains unconcerned about the labor exploitation of workers by these commercial farms, although it monitors the nature of the mobility and maintains some demographic data of the migrants.

The problem of labor exploitation by both domestic and foreign investors is also linked to the way the government has promoted the abundance of land and labor, as well as spectacularized the supposed investment bonanza in the country. In line with this, a top official at the Oromia investment and industry bureau has clearly stated what he perceives as the weakness of the government as follows:

*To attract investors from around the world and promote investment opportunities in our country, we declared at the outset that we had cheap labor and empty land as incentives. That was a big mistake. Instead of portraying our resources as cheap, we should have promoted their abundance and high good quality. Thus, we are accountable for creating such an environment for the investors to exploit labor and other resources.*

Similarly, the interviewed investors also indicated that most of the local people are not willing to work as laborers in their farms, though they want to prioritize the locals for these employment opportunities as stated in the investment proclamation. These investors, therefore, prefer to hire migrant farmworkers for cheaper wage payment. As noted by Li (2011), the logic of investment always follows the logic of capital that capital always goes to where it generates profit. Therefore, in whatever arrangement, investors always look for “cheap, abundant and disciplined labor” in order to get profit from the production of crops (Li 2011, p. 288). One of these investors explained that “*If there was no access to such farmworkers coming from our neighboring regions, our investment in these farms would not have been possible since we mainly depend on human labor force and do not own advanced farming technologies*.” Throughout the world historically, owners of big capital often prefer to hire migrant workers because they are usually cheaper than local workers, and above all, they are easy to control socially and politically, especially when they are to work from one enterprise to another, and where contracts are not always legal or formal (Ngai 2014; McWilliams 2000). Advocacy groups, including trade unions, have difficulty organizing this type of labor (Shah and Lerche 2020).

In Oromia, the small-/medium-scale investors hire the migrant workers on a daily wage basis, mainly during the harvesting season and for various tasks at different stages of farming, including plowing, planting, wedding, spraying chemicals, loading, and so on. These investors said there is no formal labor agency that recruits and supplies these workers for them. However, there is a specific place in one town where they pick up the migrant workers. Only a few of the interviewed investors mentioned that they have informal brokers who recruit these daily laborers from the town and bring them to the farms. Some others also mentioned that they receive the mobile number of those workers who demonstrated good performance during their first hired day, and then they call them for the next working days. Most of these small-scale investors hire on average about 30 to 40 migrant daily laborers during the peak season of farming, which is most often for about 6 months. The investors pay 6–7 USD per day for a daily laborer, which they consider a fair wage. However, the workers claimed that it is too low to support their livelihood. When asked to evaluate the amount they earn form working as a daily laborer in these investment areas, about 81.6% of the migrant workers who participated in the survey indicated that it is very low (See Table 1).

**Table 1:** Amount of the wage obtained from labor work, as perceived by the migrant workers

Wage from labor work	Number of cases	Percent
Very high	0	0.0
High	0	0.0
Fair	5	4.2
Low	17	14.2
Very low	98	81.6
Total	120	100.0

The exploitation of migrant labor by these emerging agrarian capitalists has gender and generational dimensions (Ossome 2022; White 2020), where female workers are more exploited than their male counterparts. Though both males and females are working as daily laborers in these farms, they are not equally treated when it comes to wage payments. The female workers claimed that they receive significantly lower wage payments compared to males, even for the same tasks. It is widely perceived that male workers work harder and can deal with more challenging tasks than females. As a result, they obtain about 6 USD per day, while female workers receive 4 USD per day. In general, these migrant workers expressed their dissatisfaction with their current jobs on these farms since they are unsustainable, tiresome, and harmful, in addition to low wage payments. Those workers who participated in the survey reported that they work as daily laborers in these investment areas, on average, for 124 days annually. As reported by the interviewed investors, most of them hire only a few workers, not more than five, based on a monthly contract to work as irrigation machine operators, guards, and drivers. These workers are employed for about six to seven months, with a monthly salary of 80 to 100 USD depending on the nature of task they work.

The 120 surveyed migrant workers (82 males and 38 females) were also asked why they decided to leave their communities. Most of the factors mentioned by the respondents were directly or indirectly related to land. As indicated in Table 2, while landlessness was reported by the majority of the respondents (87%), other factors, including small farmland size, low income, decreased agricultural productivity, and shortage of food, were also mentioned as important factors, which are also related to land issues in various ways. The migrant workers were also asked if they had any jobs back in their villages. While 76% of them reported that they didn’t have any job, 24% mentioned that they had a job with very meager wage income, which was also not sustainable. During an in-depth interview, one of these migrant workers who do not own land back in his village explained his situation as follows:

*Since I do not have land back in my village, I decided to migrate looking for a job here in the farm investment areas. My parents own a farmland, which is too small to produce adequate food for the family. Therefore, they did not give me any piece of land that I could use for myself. I was not granted land by the government since I was too young to be considered for land allocation during the last land redistribution, which was conducted three decades ago. At that time, I was under eighteen and hence not eligible to obtain land as per the constitution of the country.*

**Table 2:** Factors for outmigration of migrant farm workers

Reasons to migrate	Number of cases	Percent
Landlessness	104	87.0
Small farmland size	16	13.0
Decreased agricultural productivity	86	71.6
Shortage of food	75	62.5
Did not have any job	91	76.0
Had a job but with too low income	29	24.0

During the focus group discussion with the migrant workers, participants also explained that the primary reason they left their village was shortage of farmland. According to them, they are currently facing many challenges, including ethnic-based discrimination as well as lack of accommodation, which exposes them to various health risks and criminal attacks. As a result, they would prefer to go back to live with their families if they were granted farmland in their communities. In line with this, a participant explained why he migrated as follows.

*I used to live with my parents, farming their land and producing food for the family. But now I am 26 and hence too old to continue living with my parents. I want to have an independent life. I want to marry and establish my own family. However, I do not have my own land to produce enough for myself. So, I decided to come here and see if I can save money from wage work in these investment areas. Indeed, we are far away from families, and the condition here is not good. We are here in a different ethnic group, dealing with social discrimination and other problems. If I get my own land, I will quit working here and go back to my home immediately.*

Those migrant workers who own land in their home villages (16 participants) claimed that it is too small to produce enough to feed their families. The average farmland size owned by these migrants is 0.45 hectares. They were also asked about what happens to their land in their absence while migrating to other areas for wage labor. Some of them mentioned that their spouses and/or other relatives manage their land on their behalf. Others also stated that they contracted out their land to others in the community either for cash or share cropping. Many of them also said that they go back to their home when there is no work in these investment areas and stay working on their own farms until they return to these areas during the next working season.

The seasonal mobility of these migrant workers depends on the agricultural calendar, switching between agricultural peak period (June to November) when the major agricultural activities are undertaken and agricultural slack period (December to February). About 71% of the participants mentioned that they, and some members of their families, sometimes migrate to urban centers such as Adama, Awassa and Addis Ababa to work in other sectors, including construction, manufacturing, and service provision. They go to these areas during the agricultural slack period, then return to these investment farms during the next farming season. In line with this, a 24-year-old female migrant worker describes how she continues working across different sectors as wage laborer during different seasons.

*I came from Wolayta to work as wage laborer on these farms here. This is my third time to be here since 2021. Every time I come here; I stay for about four to five months working in these farms. When the farming season ends, I do not go back to my home because there is no job there. I may go there just for a few days to visit my family. During these seasons, I usually go to Addis Ababa to work as domestic servant or waitress in restaurants. Since the wage here is relatively better than what I earn from working in the cities, I come back here to work in the farms during the faming season.*

These migrant workers described that they also sometimes go to other nearby regions, mainly Gambella region, where there are such commercial farms that require intensive human labor. Access to information regarding employment opportunities in these commercial farms outside their region was very crucial for them to reach a decision to migrate to these areas. About 62% of the 120 respondents indicated that they got such information from relatives and family members, and 76% of them from friends who already migrated to these areas. To get in contact with these sources of information, most of them use their own channels of communication or meet these migrants in person when they are back to their village to visit their family. The flow of information and personal networks are, therefore, very crucial for migration decisions by these laborers, as most of them made their decision in consultation with other family members and friends (See Table 3).

**Table 3:** Source of information about employment opportunities in the destination areas

Source of information	Number of cases	Percent
Relatives/family members who have migrated	74	61.7
Friends who have migrated	91	75.8
Radio/Television news	32	26.7
Government agency	2	1.7
Social media	23	19.2

Participants were also asked what they do with the income they obtain from working as daily laborer on these commercial farms. 85% of them reported that they send money to their home every month to support their families with various types of social reproduction needs, mainly to cover expenses for food, medical care, clothes, and school fees for children. Although most of them intended to save money to start their own productive business, like opening shops and restaurants, they could not make it since they were continuously sending money to their families. Only 15% of the respondents reported that they were able to save money from their income, which they used to buy cattle, rent land for farming, and start small business of their own. 89% of the respondents explained that most of the time their household does not have savings and there are even more times that they are in debt, borrowing money for food from relatives, friends, and private lenders. As a result, the money these migrant workers send back home partially used to repay debts of credits from microfinances and private lenders that they took when they face financial crisis.

Labor migration in the context of rural societies in Ethiopia has also significant implications for the social reproduction tasks of household members. Women in Ethiopia generally tend to be exclusively responsible for most of the social reproduction tasks, including cleaning, taking care of children and other members of the family, managing small animals and dairy products, and some farming activities only in their areas. Women’s involvement in other income generating activities and their physical mobility to other areas is basically constrained by cultural and religious norms, which require them at least to obtain consent from their husbands (Yisehak 2008; Waithanji et al. 2013). Accordingly, the social reproduction tasks in migrants’ households are mainly managed by the female members, who often stay at home while the men do migrate for work and send money back to their family. Table 4 shows that while 47.6% of male migrants were married and have left their wives and children at home, none of the female migrants were married, with 34.2% divorced and 65.8% never married yet. Moreover, these married male migrants who own land are compelled to leave their wives at home due to the rural land policy, which expropriates land holders if they stay away from their kebele for more than six months. Some of these married male migrants explained that they let their wives stay at home partly to take care of their farmland so that they would not be expropriated by the government.

**Table 4:** Sex and marital status of the sampled migrant farm workers

Marital status	Male		Female		Total	
	Number	Percent	Number	Percent	Number	Percent
Married	39	47.6	0	0.0	39	32.5
Never married	32	39.0	25	65.8	57	47.5
Divorced	11	13.4	13	34.2	24	20.0
Widowed	0	0.0	0	0.0	0	0
Total	82	100.0	38	100	120	100.0

As discussed above, majority of those who migrate are men. While large-scale investments cause outmigration from the Upper Awash Valley, the expansion of small-/medium-scale commercial farms in these areas leads to the immigration of farm workers from other regions. Such migration of the male productive forces, in both cases, is mainly caused by diminished access to land, leading to a reallocation of labor among household members.

In the Upper Awash valley, the outmigration of men, mainly due to the impact of large-scale investments, has forced women to take on more social reproductive tasks. Some of them have started working as daily laborers on the investment areas, as well as engaging in other income-generating activities to fill the gap created due to the absence of male household members. The loss of communal grazing land due to large-scale land transfers has compelled villagers to drastically reduce their number of livestock, leading to a significant cut in the production and sale of dairy products. This has significantly affected women since dairy products were their most important source of income. They used such income to purchase food and other necessities for their family members, including clothes, medical expenses, and school fees for their children. Such significant decrease in the income of women has, therefore, compelled them to look for additional and new sources of income generating activities. In line with this, one of the participants of a focus group discussion conducted in in the study kebele explained her situation as follows:

*Previously, we had access to adequate grazing land and water sources in our community. We are displaced not just from our lands, but also our access to the nearby River is significantly diminished, as it is almost exclusively used by these investment farms. We used to depend on this river to water both our animals and farmlands. Hence, we had many cattle, and we used to produce much milk and butter for our own consumption as well as to sell in the market to earn cash income. That grazing land is now given to a big flower farm, preventing us from accessing it to graze our animals. Consequently, we have sold most of our animals and currently we just have one cow, and two oxen left.*

As argued by Shattuck and colleagues, “environmental conditions contribute to the burden of social reproductive labor” (2023, p. 503). The environmental consequences of both the large-scale and small-scale commercial farms in the Upper Awash valley also have significant consequences on the labor burden of women within the affected households. Other participants in the discussion added that the grazing areas and forests that are taken by both large- and small-scale investors were also nearby source of firewood for the community people. According to them, women in the community are currently forced to travel to remote areas to get firewood, or they should buy it from the market since it is still the main fuel for cooking. They also mentioned that they are not earning cash income any more from the sale of the firewood, which they used to collect from these communal areas. Similarly, the outmigration of males from the neighboring regions to the upper Awash valley of Oromia region have caused women to be in charge of much more reproductive tasks during the absence of their spouses. This is mainly due to the fact that they started to take over reproductive activities in the communities, beyond the domestic chores and biological reproductive roles in their households. Interviewed male labor migrants, who left their wives and children at home, confirmed that their absence has significantly increased the workload of their wives, requiring them to engage in additional burden of the daily routines that were previously handled by the male members. For example, some of them have become fully in charge of managing agricultural land and production; others have started to engage in other income-generating activities such as petty trade in their communities in addition to dealing with all the household responsibilities and raising children alone in the absence of their spouses.

## Conclusion

This paper shows that the global land rush has created an opportunity for capital accumulation, not only from above by large-scale corporate level capitalists, but also through a different route of accumulation by aspiring agrarian capitalists from below. The classical understanding of the emergence of social differentiation-induced sector of capitalist farmers and entrepreneurs is that land and capital accumulation emerge from within the agriculture sector. But the Ethiopian case shows a route where individual entrepreneurs from the rural, but mostly from the urban centers, have emerged to become the overwhelming drivers of land and capital accumulation through small- and medium-scale capitalist farming and other complementary entrepreneurial investments.

This land dynamics emerging from out of the land rush cannot take the shape it has taken if not for the changing dynamics of labor regime that the land rush itself has reshaped. This can be seen in two ways. On the one hand, the capital-intensive large-scale commercial farms have displaced smallholders without providing significant job opportunities, causing outmigration from the land grab sites. Since they consider the wage is too low in these emerging small/medium-size capitalist farms, the local Oromia working people do not want to take these emerging jobs and prefer to migrate to other industrial centers and urban areas searching for better payments, leading to a labor shortage in these areas. On the other hand, the flourishing of labor-intensive small-/medium-scale commercial farms has resulted in a massive labor immigration from other neighboring regions into the land grab sites. The land rush and the availability of cheap migrant labor from the poorer regions of Ethiopia have, therefore, contributed to the significant expansion of small-and medium-scale commercial farms by emerging capitalist farmers and entrepreneurs.

Ultimately, this paper shows the inseparability, empirically and analytically, of land and labor, as well as production and social reproduction dynamics in explaining the character and trajectory of social change. This insight is also underscored in some studies of similar farmer-migrant farmworker interlinkages, although involving international migration (Borras et al. 2022b; Mortensen 2024; Xu et al. 2025). The role played by migrant labor has been manifested in two interconnected ways underpinned by a singular logic and social force, that is, the capitalist commodification of land and labor, namely, outward labor migration away from the land grab sites, and the inward migrant labor inflows into the same land grabs sites.

## Footnotes

1 By land regime, I mean the pattern of “interconnections between land-based social structures, formal and informal institutions that enable, provide and maintain the scaffolding for such structures, and the correlation of state and social forces and political power that legitimize this structural and institutional order” (Borras and Franco 2025a, p. 72).This restructuring would have a substantial impact on power dynamics within rural communities, leading to political implications at the regional, national, and international levels.

2 Kebeles are forms of neighborhood associations that are the smallest administrative units in the Federal Democratic Republic of Ethiopia (FDRE). They are accountable to their respective woredas, and they have their own local government consisting of a chairperson, an elected council, a cabinet, a social court, and development as well as security staff such as police/militias.

3 Woredas, also called districts, are the third level of administrative division of the FDRE, next to regional states and zones, respectively.

4 The Oromia regional state adapted a Regional Rural Land Administration and Use Regulation No.151/2012 for implementation of Regional Rural Land Administration and Use Proclamation No. 130/2007.

5 Central Ethiopia Regional State is a recently established region, splitting from the SNNP region in August 2023. The fieldwork to collect the data for this study was conducted in March-April 2023, which was before the establishment of this region.
